# Assessing the Feasibility of Visual Charts to Augment Diarrheal Disease Prevention Practices: A Qualitative Study with Caregivers in Kenya

**DOI:** 10.24248/eahrj.v9i2.846

**Published:** 2025-12-24

**Authors:** Frida A. Okeyo, Edna Nyangechi, Bernard Guyah

**Affiliations:** a School of Public Health, Maseno University, Kenya

## Abstract

**Background:**

Diarrhoeal disease remains a leading cause of morbidity and mortality among children under five, particularly in low-income urban settings. Despite interventions such as rotavirus vaccination, vitamin A supplementation, improved hand hygiene, and water, sanitation, and hygiene (WASH) initiatives, diarrhoeal episodes persist. Visual charts offer a potentially effective educational tool for low-literacy populations. This study explored the feasibility and acceptability of using visual charts to support diarrhoeal disease prevention practices among caregivers in Nyalenda, Kenya.

**Methods:**

A qualitative descriptive study was conducted using in-depth interviews with 14 caregivers of children aged 6 to 24 months and key informant interviews with 2 community health assistants and 2 health facility in-charges. Participants were purposively sampled based on child age, recent diarrhoeal episodes, and residence stability. Interviews were audio-recorded, transcribed, and thematically analysed.

**Results:**

Caregivers had considerable knowledge of diarrhoeal disease and prevention, but practices were inconsistent due to economic and environmental constraints, including limited soap and safe water access, unimproved sanitation, and congested living conditions. None of the participants had prior exposure to visual charts. All caregivers and health providers expressed strong interest in using visual charts, suggesting content on handwashing, water treatment, food hygiene, and solid and liquid waste management, with materials in English, Kiswahili, and Luo. Charts were envisioned as household reminders and training aids.

**Conclusions:**

Visual charts were well-received and considered feasible to support diarrhoeal disease prevention in resource-limited settings. Findings highlight high receptivity and potential for future interventions to enhance knowledge and adherence to preventive practices.

## BACKGROUND

Diarrhoea is the second leading cause of mortality among children globally.^1^ The World Health Organization (WHO) defines diarrhoea as 3 or more loose stools per day or a more frequent passage than usual.^2^ It is primarily caused by ingestion of pathogens via unsafe drinking water, contaminated food, or unclean hands. Poor food preparation, storage, and dirty utensils also contribute to diarrhoea.^3–6^

Annually, 1.7 billion cases of childhood diarrhoea occur globally^2^, causing around 525,000 deaths among children under five.^7^ Africa and Asia account for nearly 80% of these deaths.^8–9^ In Kenya, the 2015 Demographic and Health Survey reported a 15% prevalence among under-fives.^10^ Kisumu, in the Nyanza region, is among the highest-burden areas (18–20%).^8^ Diarrhoea results in an estimated 1,140 Disability-Adjusted Life Years (DALYs) per 100,000 children and economic losses of ∼1.14% of Kenya's GDP.^11^

Despite interventions such as rotavirus vaccination, improved water quality, promotion of exclusive breastfeeding, vitamin A supplementation, and handwashing campaigns^12–20^, diarrhoeal disease remains prevalent, particularly in children aged 6 to 24 months. Knowledge alone does not always translate into practice. Visual aids have been shown to enhance comprehension, recall, and adherence, especially in low-literacy settings.^21^ Visual charts make information accessible, memorable, and understandable across languages, potentially bridging gaps in household-level prevention.^22–23^

This study aimed to assess the feasibility and acceptability of using visual charts to augment diarrhoeal disease prevention practices among caregivers of children aged 6 to 24 months in Nyalenda, Kenya.

## METHODS

### Study Design

This qualitative descriptive study was part of a formative phase to inform interventions for reducing diarrhoeal disease among children aged 6 to 24 months. It involved In-Depth Interviews (IDIs) with caregivers whose children had experienced diarrhoea within the previous 3 months, and Key Informant Interviews (KIIs) with health personnel, focusing on feasibility and acceptability of visual charts.

### Study Setting

The study was conducted in Nyalenda A, an informal settlement of Kisumu City with a population density of 5,317 people/km^2^ and widespread low-income housing. The site was selected due to a high burden of childhood diarrhoeal disease.

### Participants

Eighteen participants were purposively selected, comprising 14 caregivers and 4 key informants (2 facility in-charge, 2 community health assistants). Eligibility criteria included residing in the study area for at least 3 months, having a child aged 6 to 24 months, and a recent history of diarrhoeal episodes. Confidentiality was maintained through anonymization of participant identifiers and secure storage of audio recordings and transcripts. Owing to the qualitative study design, external validity is inherently limited. However, the findings remain transferable to comparable urban informal settlement contexts.

### Procedures

Data were collected using semi-structured interview guides in English, Kiswahili, and Luo. Interviews were conducted in person or by phone according to participant's preference. Two trained research assistants carried out the interviews after undergoing a three-day training. A pilot test with two caregivers informed final tool refinement. Probing questions (“What?”, “Why?”, “How?”) ensured depth of data. Saturation guided sample size.

### Data Analysis

Interviews were audio-recorded, transcribed, and read multiple times by the research team. Codes were applied manually, organized in a matrix, translated into English, and grouped into categories and subcategories. Thematic analysis was conducted using Excel. Reporting was guided by the qualitative research review guidelines (RATS).^24^

### Ethical Considerations

Ethical approval was obtained from Maseno University Scientific and Ethics Review Committee (Approval number: MUSERC/01208/23) and the National Commission for Science, Technology & Innovation (Licence Number: NACOSTI/P/23/27423). Verbal informed consent was obtained. Participant confidentiality and anonymity were maintained.

## RESULTS

### Current Practices and Gaps

Water treatment: Half of the caregivers (n=7, 50%) reported boiling drinking water, although only 2 (14%) did so consistently. Water storage practices varied, with 8 caregivers (57%) keeping water covered.

Food hygiene: Half of the caregivers (n=7, 50%) ensured thorough boiling of food; while the relied on subjective judgement of readiness. Most (n=9, 64%) did not cover food during storage. Vegetable washing practices varied; 10 (71%) washed the vegetables after cutting them.

Handwashing and hygiene: While all caregivers could identify critical times for handwashing, only 4 (29%) consistently used soap. Kitchen cleaning, utensil hygiene, and waste disposal were inconsistently practiced.

Caregiver perspectives reflected financial and environmental Constraints, as quoted by participants:

“*In as much as I am aware that I should boil or treat my drinking water, sometimes I lack money and I'm forced to drink directly from the tap.”*

“*Given the high cost of life, it is now difficult for me to buy enough water; sometimes I even keep my child's soiled clothes until I get money to buy soap.”*

### Caregiver & Health Worker Receptivity to Visual Charts

None of the participants had prior experience with visual charts. All expressed interest in using them at household level to reinforce preventive practices. Participants suggested charts covering handwashing, water treatment, food hygiene, solid and liquid waste management, with materials in English, Kiswahili, and Luo.

Participant quotes:

“*I will be glad to be introduced to visual charts; I hope you will provide copies to carry home so I can train my older children or other household members*.” Caregiver

“*Use of visual charts is an idea that has not been utilized in this area. If you can design the charts in a language well understood by caregivers and covering all areas of diarrhoeal disease prevention, it will be very useful.”* —Health center in-charge

## DISCUSSION

This study explored the feasibility and acceptability of visual charts to augment diarrhoeal disease prevention practices among caregivers of children aged 6 to 24 months in an informal settlement in Kisumu, Kenya. Our findings highlight 3 critical areas: persistent gaps in current practices, contextual barriers to effective prevention, and high receptivity to visual charts as a novel educational tool.

**Current Practices and Knowledge Gaps:** Caregivers demonstrated substantial knowledge of diarrhoeal disease prevention, including the importance of handwashing, safe water handling, and hygienic food practices. However, knowledge did not consistently translate into practice. Water treatment and storage practices were inconsistent, with only a minority of caregivers boiling water regularly. Food hygiene practices varied, with many caregivers unable to consistently cover food or wash vegetables before cutting. Handwashing was acknowledged as critical at key times, but regular use of soap was limited. Solid and liquid waste management practices were suboptimal, reflecting environmental constraints such as shared sanitation facilities, lack of drainage, and congested living conditions. These findings align with prior studies in similar low-resource urban settings, which report a persistent “knowledge–practice gap” where caregivers understand preventive behaviours but face challenges in implementation.^25–28^

**Contextual Barriers to Practice:** Economic and infrastructural constraints emerged as the main barriers to effective diarrhoeal disease prevention. Limited financial resources restricted caregivers’ ability to purchase soap or fuel for boiling water. High population density, shared sanitation facilities, and inadequate drainage hindered safe waste disposal. Such environmental constraints are consistent with evidence showing that informal settlements disproportionately expose children to enteric pathogens.^8^ These structural barriers underscore the need for interventions that are low-cost, contextually appropriate, and feasible for household-level adoption.

**Feasibility and Acceptability of Visual Charts:** Visual charts were universally welcomed by caregivers and health workers, who perceived them as practical, easy-to-understand, and memorable tools for reinforcing preventive behaviours. Participants suggested that charts should cover handwashing, water treatment, food hygiene, and solid and liquid waste management, with materials in English, Kiswahili, and Luo. Importantly, caregivers envisioned using charts at home as reminders and training aids for older siblings or other household members. These findings support previous evidence that visual aids improve comprehension, retention, and adherence to health recommendations, particularly in low-literacy settings.^21–23^

Visual charts have the potential to address several barriers identified in this study. By providing a constant, visible prompt, charts can help mitigate forgetfulness or inconsistent adherence due to competing daily demands.^21^ The use of culturally appropriate and multilingual visuals may overcome literacy and language barriers, enhancing equitable access to health information.^22–23,29^ Moreover, charts can complement existing health education efforts by reinforcing messages delivered during health facility visits or community health outreach.

### Implications for Intervention Design

Our findings have important implications for design of household-level diarrhoeal disease prevention strategies. First, interventions should account for contextual barriers such as limited soap access, shared sanitation facilities, and economic constraints. Visual charts could therefore be complimented with low-cost. practical solutions like affordable soap substitutes, water purification methods, and improved storage containers Second, developing visual charts through a participatory approach, with caregivers contributing tocontent, language, and layout, may enhance acceptability and cultural relevance. Third, integrating chart use into caregiver training and encourage sustained practice.

Visual aids have been successfully used in diverse health and safety contexts, including food safety, infection control, and immunization promotion.^30–33^ Studies indicate that visual materials can enhance understanding, improve recall, and motivate behaviour change, particularly in populations with low literacy or limited prior exposure to formal health education. Our study adds to this evidence by demonstrating that caregivers in resource-limited urban settlements are receptive to visual charts for diarrhoeal disease prevention and can envisage practical ways to integrate them into daily routines.

### Strengths and Limitations

This study provides rich qualitative insights into caregiver perceptions and the feasibility of using visual charts in a low-resource urban context. The use of purposive sampling and inclusion of both caregivers and health workers allowed triangulation of perspectives. However, the study has limitations. The sample was small and drawn from a single informal settlement, limiting generalizability. Self-reported practices may be subject to social desirability bias. Additionally, the study assessed feasibility and acceptability, but not actual behavioural change; future research should evaluate the effectiveness of visual charts in improving diarrhoeal prevention behaviours and reducing disease incidence.

## CONCLUSION

Overall, our study highlights persistent gaps in diarrhoeal disease prevention among caregivers in Nyalenda, driven by structural and economic barriers. Visual charts were well-received and perceived as a feasible, low-cost tool to support adherence to preventive practices. By making information visible, accessible, and memorable, visual charts have the potential to complement existing health education strategies and strengthen household-level disease prevention efforts in low-resource urban settings.
